# Supplementary material for: Localization of muscarinic acetylcholine receptor 2 to the intestinal crypt stem cell compartment
Source: Data Brief. 2016 Dec 21;10:482–6. doi: 10.1016/j.dib.2016.12.032 (PMC5196237; doi:10.1016/j.dib.2016.12.032)
Supplement: Supplementary file 1 — Supplementary material [file mmc1.doc]

*Data article*

**Title: *Localization of muscarinic acetylcholine receptor 2 to the intestinal crypt stem cell compartment***

**Authors: Eleanor D. Muise, MD, Neeru Gandotra, PhD, John J. Tackett, MD, MHS, Michaela C. Bamdad, BA, and Robert A. Cowles, MD**

**Affiliations: Department of Surgery, Section of Pediatric Surgery, Yale School of Medicine**

**Contact email: robert.cowles@yale.edu**

**Abstract**

The data presented in this article are related to the research article entitled “Distribution of muscarinic acetylcholine receptor subtypes in the murine small intestine” (E.D. Muise, N. Gandotra, J.J. Tackett, M.C. Bamdad, R.A. Cowles, 2016).[1] We recently demonstrated that neuronal serotonin stimulates intestinal crypt cell division, and induces villus growth and crypt depth.[2, 3] Scopolamine, a nonspecific muscarinic receptor antagonist, inhibited serotonin-induced intestinal mucosal growth.[2] Here we provide data regarding the localization of muscarinic acetylcholine receptor 2 to the intestinal crypt stem cell compartment.

***Specifications Table***

| Subject area | *Biology* |
| --- | --- |
| More specific subject area | *Muscarinic acetylcholine receptors* |
| Type of data | *Figures* |
| How data was acquired | *RNA extraction, RT-PCR, Immunofluorescence, Microscopy (Zeiss 510 Laser Scanning Confocal microscope, Carl Zeiss International, Germany)* |
| Data format | *Raw* |
| Experimental factors | *No pretreatment* |
| Experimental features | *Muscarinic acetylcholine receptor subtype presence determined by RT-PCR, and localization determined by immunofluorescence and confocal microscopy* |
| Data source location | *Yale University College of Medicine, New Haven, CT* |
| Data accessibility | *The data are supplied within this article* |

**Value of the data**

- **Muscarinic receptors are ubiquitous throughout the gastrointestinal tract**
- **Nonspecific blockade of muscarinic receptors blunts neuronal serotonin stimulated growth**
- **Muscarinic acetylcholine receptor subtype 2 localizes to Paneth cells within the intestinal crypt stem cell compartment**

**1. Data**

Data show all five muscarinic acetylcholine receptor subtypes (mAChR) were present in the mouse duodenum, jejunum, and ileum across all ages of mice by RT-PCR (Fig. I). Mouse ileum was further imaged with immunofluorescence microscopy and highlighted mAChR2 within intestinal crypts (Fig. II). mAChR2 was localized to the crypt stem cell compartment (Fig. III), and further co-localized within Paneth cells in the intestinal crypt with lysozyme (Fig. IV).


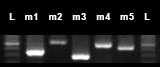


Figure I. mAChR RT-PCR. Photo of mAChRs extracted from jejunum of 6 week-old C57BL/6 mouse, mAChRs 1-5 flanked by ladder (L) in 1.5% agarose gel with ethidium bromide.


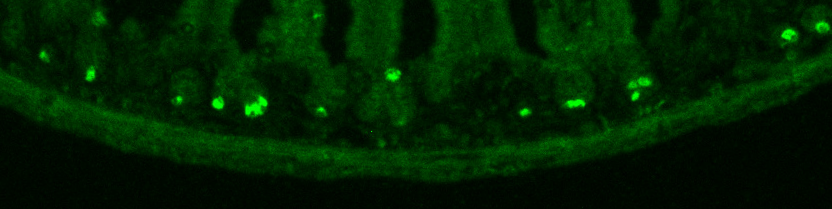


Figure II. Immunofluorescence staining for subtype specific mAChR2 localized to intestinal crypts. 10x.


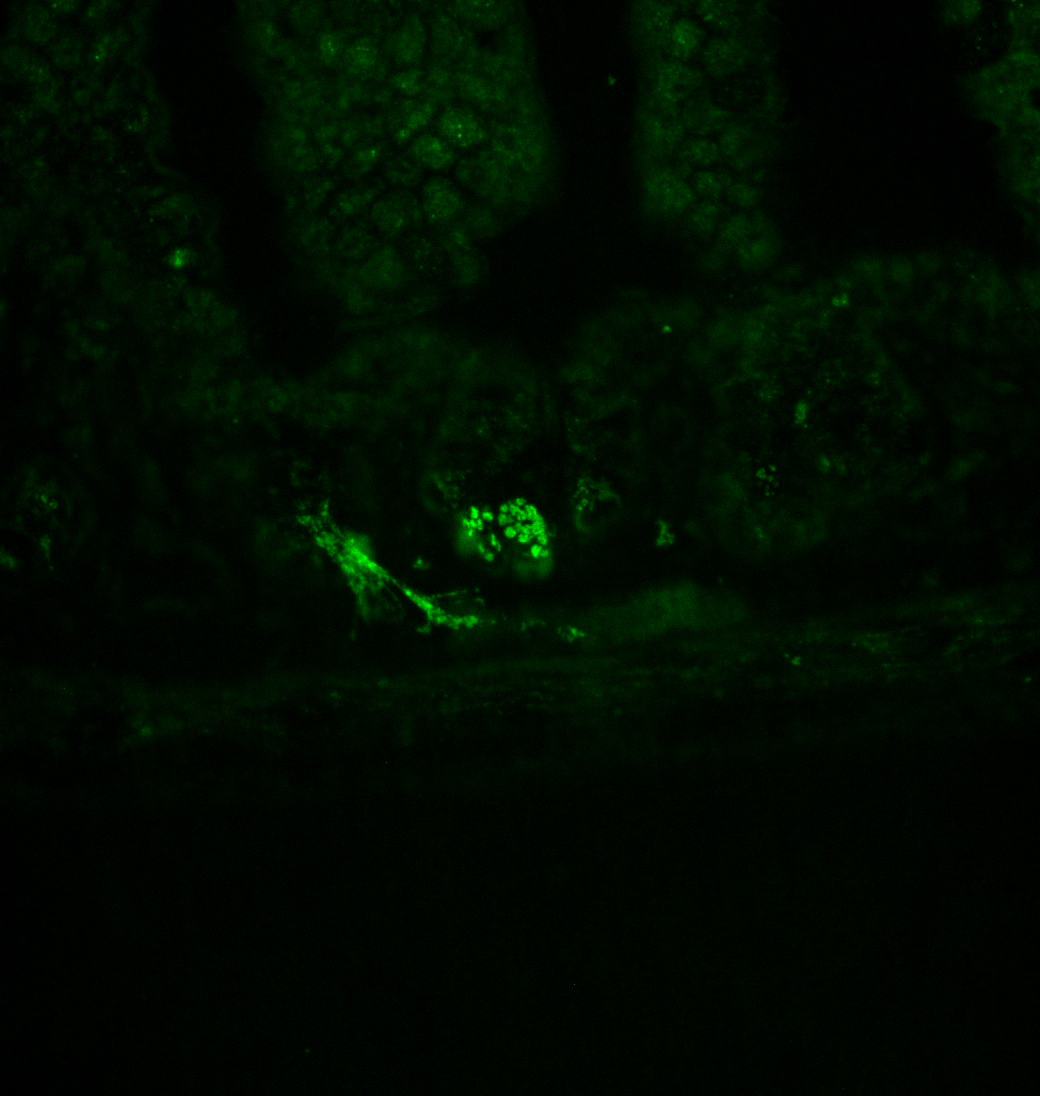


Figure III. mAChR2 localized to intestinal crypt stem cell compartment. mAChR2 labeling illuminated on channel 488 demonstrates a discrete granular pattern within the intestinal crypt stem cell compartment.


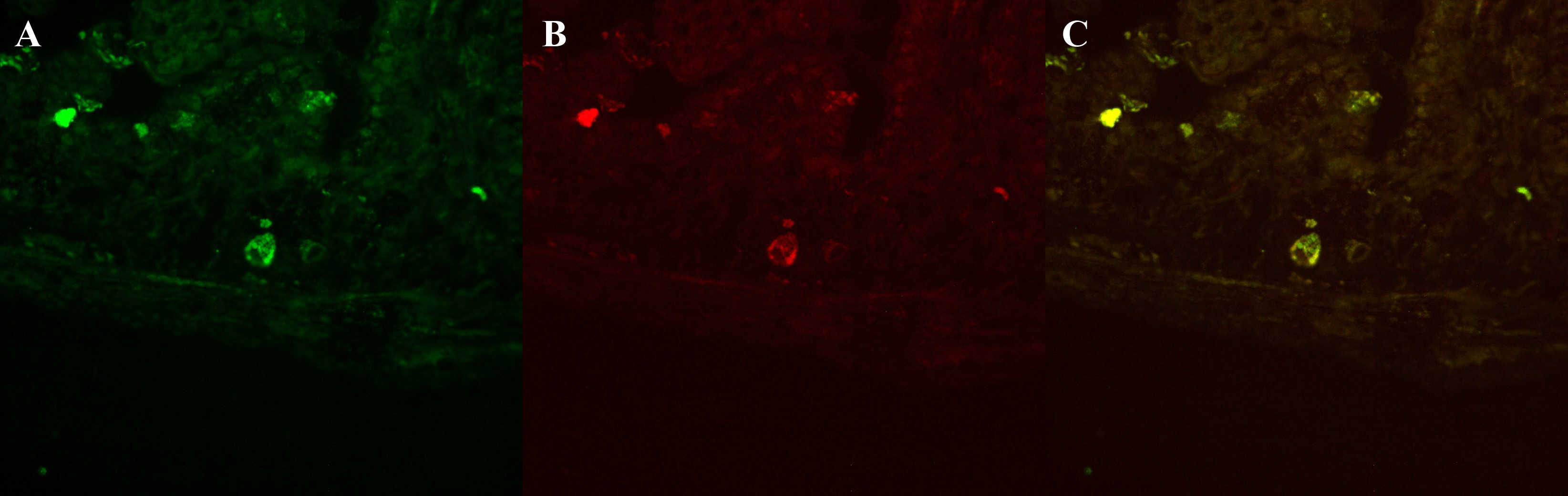


Figure IV. mAChR and Lysozyme co-localize to Paneth cells in the intestinal crypt. A demonstrates mAChR2 on channel 488 with an intracellular granular pattern in the center of the image. B captures the same slide on channel 594, stained for lysozyme, a Paneth cell marker, which highlights the characteristic granules. C illustrates the merge of both images with yellow color representing areas of overlapping signal.

**2. Experimental Design, Materials and Methods**

2.1 Animals

This study was approved by the Institutional Animal Care and Use Committee, and animals were maintained in accordance with guidelines from this committee on the care and use of laboratory animals, and described in detail previously. [1]

2.2 Real-time polymerase chain reaction (RT-PCR)

RNA isolation was performed using RNeasy Kit (Qiagen) and concentration measured using Nanodrop (Thermo Scientific). Two micrograms of RNA was used as a template for cDNA synthesis, and reverse transcription was performed with KAPA Mouse Genotyping Kit (Kapa Biosystems). Subtype specific primers for mAChR 1-5 (Table 1) were used for amplification under the following conditions: 3 min @95°C, 30sec @95°C, 50sec @60°C, 30sec @72°C x30 cycles, with 10min @72°C extension and held at 12°C. The products were separated by gel electrophoresis (1.5% agarose gel with ethidium bromide).

Table 1. mAChR subtype specific primer sequences for RT-PCR

| Gene | Sense | Antisense |
| --- | --- | --- |
| *mAChR1* | 5’-GCACAGGCACCCACCAAGCAG-3’ | 5’-AGAGCAGCAGCAGGCGGAACG-3’ |
| *mAChR2* | 5’-GGCAAGCAAGAGTAGAATAAA-3’ | 5’-GCCAACAGGATAGCCAAGATT-3’ |
| *mAChR3* | 5’- GTCTGGCTTGGGTCATCTCCT -3’ | 5’-TTCGTGCCTTGCTGTTGTAG-3’ |
| *mAChR4* | 5’-AGTGCTTCATCCAGTTCTTGTCCA-3’ | 5’-CACATTCATTGCCTGTCTGCTTTG-3’ |
| *mAChR5* | 5’-CTCATCATTGGCATCTTCTCCA-3’ | 5’-GGTCCTTGGTTCGCTTCTCTGT-3’ |

2.3 Immunohistochemistry

Ileum from 2 week-old mice was flushed and fixed with Nakane fixative, washed with tris-buffered saline (TBS), dehydrated with ethyl alcohol, and subsequently placed in propylene oxide. Fixed dehydrated tissue was embedded in epon (Electron Microscopy Sciences) then sectioned at 1-2 microns.

Etching was performed with potassium hydroxide in methanol and propylene oxide to dissolve the plastic, and subsequently rinsed with methanol then TBS. Antigen retrieval was performed with heated citrate buffer.

Slides for were quenched with ammonium chloride prior to blocking. Primary and secondary immunofluorescence antibodies for mAChR1-5 and lysozyme are given in Table 2. Slides were mounted with Vectashield (Vector Laboratories), and kept at -20°C.

**Table 2**. mAChR and Lysozyme Primary and Secondary Antibodies

|  | Antigen | Host Species | Dilution | Company |
| --- | --- | --- | --- | --- |
| Primary Antibodies | | | | |
|  | *mAChR1* | Rabbit | 1/20 | Millipore (AB5164) |
|  | *mAChR2* | Rabbit | 1/20 | Millipore (AB5166) |
|  | *mAChR3* | Rabbit | 1/20 | CosmoBio Co (YCUPSM3) |
|  | *mAChR4* | Mouse | 1/20 | Millipore (MAB1576) |
|  | *mAChR5* | Rabbit | 1/40 | Abcam (AB41171) |
|  | *Lysozyme* | Goat | 1/200 | SCBT (SC27958) |
| Secondary Antibodies | | | | |
|  | *anti Rabbit 488* | Goat | 1/200 | Life Technologies (a11034) |
|  | *anti Rabbit 488* | Donkey | 1/200 | Life Technologies (a21206) |
|  | *anti Mouse 488* | Goat | 1/200 | Life Technologies (a11029) |
|  | *anti Goat 594* | Donkey | 1/200 | Life Technologies (a11058) |

2.4 Imaging

Images were obtained with a Zeiss 510 Laser Scanning Confocal microscope (Carl Zeiss International, Germany). No spectral wavelength overlap was observed between Alexa 488 and Alexa 594 channels.

**References**

[1] Muise ED, Gandotra N, Tackett JJ, Bamdad MC, Cowles RA. Distribution of muscarinic acetylcholine receptor subtypes in the murine small intestine. Life Sci 2016.

[2] Gross ER, Gershon MD, Margolis KG, Gertsberg ZV, Li Z, Cowles RA. Neuronal serotonin regulates growth of the intestinal mucosa in mice. Gastroenterology 2012;143:408-17 e2.

[3] Gershon MD. 5-Hydroxytryptamine (serotonin) in the gastrointestinal tract. Curr Opin Endocrinol Diabetes Obes 2013;20:14-21.
